# CARING RELATIONSHIP DEVELOPMENT IN THE NURSING HOME IN SHANGHAI: A DYADIC QUALITATIVE STUDY

**DOI:** 10.1093/geroni/igz038.2587

**Published:** 2019-11-08

**Authors:** Lin Chen, Minzhi Ye, Qiang Zhu

**Affiliations:** 1 Fudan University, Shanghai, China; 2 Benjamin Rose Institute on Aging, Cleveland, Ohio, United States; 3 Fudan University, Shanghai, China, China

## Abstract

Caring relationships between older residents and nurse aides are fundamental in terms of service delivery in nursing homes. However, little is known for the nuanced dynamics of this relationship in China. The purpose of this study is to explore how caring relationships develop between older residents and nurse aides in the nursing home setting in urban China. Informed by the dyadic perspective, this study illustrates the development process and relational nuances by simultaneously eliciting residents’ and nurse aides’ perceptions. This qualitative study purposively sampled 20 matched resident-nurse aide dyads (N= 40) in a government-sponsored nursing home in Shanghai. Participants participated in semi-structured, in-depth interviews from January to June 2017. Thematic analysis was performed. The findings reveal that the caring relationship began with nursing home assignment and primarily focused on instrumental assistance. Gradually, emotional involvement grew within dyads and reciprocity emerged. Based on different dyadic perceptions, this study conceptualized four types of caring relationships: (a) parent-child alike, (b) mutually respectful, (c) solo performance, and (d) reasonably detached. The findings suggest that residents and nurse aides could have different views on caring relationships, which further influenced the relationship development. The four types of caring relationships shared some similar traits while differentiating from some of the common types of interactions found in the existing nursing evidence across the world. Chinese filial tradition also influenced the relationship dynamics.

